# Factors influencing the use of malaria prevention strategies by women in Senegal: a cross-sectional study

**DOI:** 10.1186/s12936-017-2095-2

**Published:** 2017-11-21

**Authors:** Mouhamed Abdou Salam Mbengue, Amy K. Bei, Aminata Mboup, Ambroise Ahouidi, Moussa Sarr, Souleymane Mboup, Oumar Gaye

**Affiliations:** 1 Institut de Recherche en Santé, de Surveillance Epidémiologique et de Formations (IRESSEF), Arrondissement 4, Rue 2D1, Pôle Urbain de Diamniadio, BP 7325, Dakar, Senegal; 2 Faculty of Health Sciences, School of Public Health, University of the Witwatersrand, Johannesburg, South Africa; 3 West Africa Global Health Alliance (WAGHA), Dakar, Senegal; 4 Department of Immunology & Infectious Diseases, Harvard TH Chan School of Public Health, Boston, MA USA; 5 Laboratory of Parasitology and Mycology-Le Dantec Hospital, Faculty of Medicine and Pharmacy, Cheikh Anta Diop University, Dakar, Senegal; 6 Department of Epidemiology and Preventive Medicine, University of Laval, Quebec City, Canada; 7 Westat, Rockville, MD USA

**Keywords:** Intermittent preventive treatment in pregnancy (IPTp), Insecticide-treated nets (ITNs), Pregnancy, Malaria, Prevention, Senegal

## Abstract

**Background:**

The World Health Organization (WHO) recommends the use of insecticide-treated nets (ITNs) and intermittent preventive treatment in pregnancy (IPTp) as a cost-effective intervention for the prevention of malaria during pregnancy in endemic areas. This study was conducted to investigate: (1) the extent of use of both IPTp and ITNs, and (2) conduct multinomial regression to identify factors affecting the optimal usage of IPTp and ITNs among women with a recent pregnancy in Senegal.

**Methods:**

Data was drawn from the 2013–2014 Demographic and Health Survey. A total of 4616 women aged 15–49 years old, who had a recent pregnancy were analyzed. Multinomial logistic regression model was used to assess factors associated with optimal uptake of malaria preventive strategies (both IPTp and ITN use).

**Results:**

Amongst women who had a recent pregnancy, less than half of them used ITNs (46.84%) however, 80.35% reported taking IPTp during their last pregnancy. Overall, 37.51% reported using the optimal malaria preventive strategies. Women aged 35–49 years and living in the richer or middle wealth quintile were more likely to use optimal prevention methods. Pregnant women living in Diourbel, Saint-Louis, Thies, Louga, Fatick and Matam were more likely to use both IPTp-SP and ITNs compared to those living in Dakar. Additionally, women who initiated antenatal care in at least at 6 weeks of pregnancy or who attended four antenatal visits or more were more likely to use optimal malaria preventive methods during pregnancy.

**Conclusions:**

This study has shown important factors that influence the uptake of malaria prevention methods during pregnancy in Senegal. These findings highlight the need for targeted preventive strategies when designing and implementing policies aimed at improving the uptake of these measures during pregnancy in Senegal.

## Background

Malaria is the leading cause of death in children in Africa [1]. In 2015, there were 429,000 malaria-associated deaths in the world and most of these deaths (92%) occurred in sub-Saharan Africa [1]. Pregnant women living in endemic areas are among the most vulnerable to malaria infection [2, 3]. Infection with *Plasmodium falciparum* during pregnancy is responsible for intra-uterine growth retardation which can lead to low birth weight and early infant death [3–6].

Currently, for malaria endemic areas, the World Health Organization (WHO) recommends a package of prevention methods to reduce morbidity and mortality associated with the disease [6]. Among pregnant women, the core preventive interventions are vector control through the provision and use of insecticide-treated bed nets (ITNs) and intermittent preventive treatment during pregnancy (IPTp) to prevent pregnancy-associated malaria (PAM) [6]. The combination of both prevention strategies has been found to be cost-effective and is associated with substantial reduction in neonatal mortality and low birth weight [7–9].

Senegal is one of the 43 sub-Saharan countries where malaria is endemic and represents one of the leading causes of childhood mortality and negative birth outcomes. However, average parasitaemia among children under five was 5.7% in 2008 and felt to 2.9% in 2010–2011 [10]. Between 2005 and 2008–2009, all-cause under-five mortality dropped from 121 to 72 deaths per 1000 live births [11, 12]. Additionally, decline of the number of malaria cases was observed after the nationwide implementation of rapid diagnostic test (RDTs) and treatment of malaria episodes with artemisinin-based combination therapy (ACT). Use of ITNs has been shown to reduce malaria incidence rate by 50% and mortality rates by 55% in children under 5 years in sub-Saharan Africa [13]. Senegal started the distribution of ITNs among children and pregnant women in 2003 and since 2011, the nationwide distribution of ITNs is extended to the general population [14] through different channels, such as health centres, community-based organizations, schools, and social marketing activities. In 2016, there was a nationwide distribution campaign in Senegal that resulted to more than 8 million ITNs being distributed across the country [15].

In addition to ITNs, IPTp with sulfadoxine–pyrimethamine (SP) has been shown to be an effective method of preventing malaria in pregnancy. The WHO recommends that IPTp-SP should be given at each scheduled antenatal care visit, starting as early as possible during the second trimester [9]. Since 2003, IPTp-SP has been available at no cost at all the public health facilities in Senegal. Following WHO recommendations in 2013, the country has transitioned from the standard 2-dose regimen to the three dose IPTp regimen, beginning in the second trimester and with treatments spaced at least 1 month apart intervals [15, 16].

In working to achieve malaria elimination, the NMCP in Senegal has outlined ambitious goals with a target of 80% of all pregnant women using ITNs and at least 80% receiving IPTp to move Senegal toward the goal of pre-elimination by 2020 [15]. There has been no recent study [17] conducted on use of malaria preventative measures in pregnant women in Senegal using a nationally representative dataset such as the Demographic and Health Survey. In this context, this study used a nationally representative dataset to investigate the factors contributing to the suboptimal uptake of prevention measures (IPTp and ITNs) among women with a recent pregnancy in Senegal to better understand the factors that might improve the implementation and scale-up of malaria prevention methods during pregnancy and to meet national and international targets.

## Methods

### Data source

**Fig. 1:**
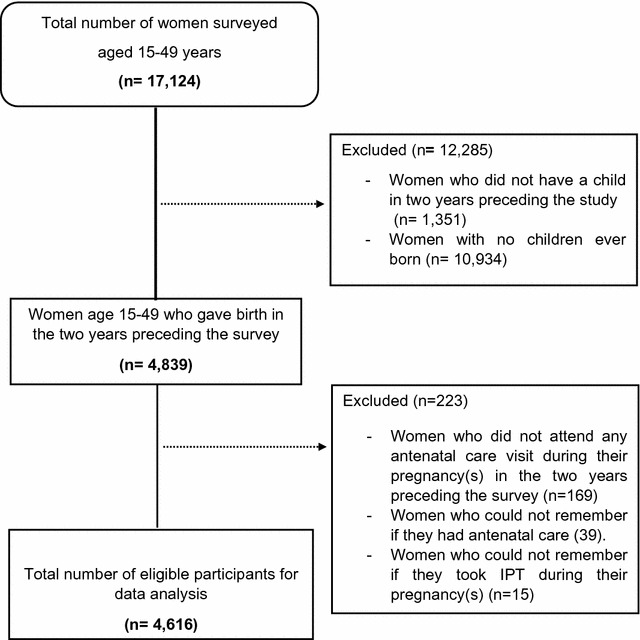
Survey enrollment flow-chart

Our data are drawn from the Demographic and Health Surveys (DHS) carried out over a period of 2013–2014 in Senegal [18]. The objectives, organization, sample design of the DHS have been described elsewhere [19]. Briefly, DHS is a nationally representative household survey with a two-stage stratified cluster sampling design. In the first stage, the primary sampling units (PSUs), which are the census districts, are selected with probability proportional to the PSU population size. At the second stage, households are selected and enumerated within each area segment. The sample was stratified by urban and rural areas. The DHS study covered 17,124 women aged 15–49 years old with a response rate of 98.7% [18]. The inclusion and exclusion criteria used to determine the eligible populations are outlined in Fig. 1.

### Study population, measurement and data management

The present study focuses on currently married women who had at least one live birth during the 2 years preceding the survey. For the most recent birth in the 2 years preceding the survey, the women were asked whether at any time during their last pregnancy they took IPTp. The response variable was limited to either those who received IPTp (yes) or who did not receive IPTp during pregnancy (no). Additionally, women were asked the question, “Did you sleep under a mosquito net the night before the survey?”. The answer to this question was assumed to represent the usage of ITNs during pregnancy. The initial variable of ITN usage consisted of four categories: “no bed net”, “only a treated net”, “treated and untreated net”, and “only untreated net”. Therefore, a new variable for ITN usage was created to limit the categories to “no bed net” and “treated bed net” and was recoded as a binary (yes/no) variable.

In order to better capture the different levels of combined uptake of ITNs and IPTp, an outcome variable with three categories was created: “none” if neither SP dose nor ITNs was used during pregnancy, “partial” if only one prevention method was taken during pregnancy, and “optimal” use of malaria prevention methods if the women reported using both methods during pregnancy. Covariates included education level, age, area of residence, provinces, maternal occupation, household wealth-index, pregnancy intention, timing of antenatal care, and number of antenatal care (ANC) visits during pregnancy. The household wealth-index is a measure of household’s standard of living and is based on data on household’s ownership of durable goods, dwelling characteristics, water sources, toilet facilities and other characteristics that are indicative of household socioeconomic status [20, 21]. For example, information on household’s good such as availability of electricity, type of water supply, type of toilet, flooring materials and number of people per room and type of cooking fuel are collected during interviews and each of these goods are assigned a weighted score generated from the principal component analysis. Finally, each household is assigned a total weighted score and the sampled population is divided into five groups quintiles ranging from 1 (the poorest) to 5 (the richest) [18–22].

### Statistical analysis

The analysis is based on data from the Demographic and Health survey conducted in 2013–2014. The analysis included the 4616 women in the age of 15–49 years who had at least one live birth. Frequency distribution (one-way tabulation) of participants across background characteristics was calculated for all variables and outcomes. Bivariate analysis was performed in the main outcome variable was cross-tabulated against each independent variable. The association between each pair of variables was tested using Pearson’s Chi square and the degree of complete use of malaria prevention methods compared across each exposure variables and women’s characteristics.

The outcome variable “uptake of prevention measures” was categorical in nature with three categories namely: “either received IPT or ITNs”; “combined uptake of ITNs and IPTp”; and “neither SP dose nor ITNs used during pregnancy”. The disadvantage of limiting the analysis to binary when variables with more than three categories has been collected is loss of detail in describing the outcome of interest and this may in turn affect the conclusion made about the exposure–outcome relationship [23]. Thus, to better understand the effect of socio-economics and demographic characteristics on optimal use of malaria prevention during pregnancy a multinomial logistic regression analysis was carried out.

To model the exposure-outcome relationship, all the variables significant at 0.25 level were included in the bivariate analysis. Any variable having a significant univariate test at 0.25 level was selected for the analysis. This significance level was selected because more traditional levels such as 0.05 can fail in identifying variables known to be important [24].

In the multivariate analysis, the outcome category “none” (neither IPTp nor ITNs were used during pregnancy) was made as baseline/reference category and the log-likelihood ratio test to select the independent variables for the multivariate model was used. The selection of independent variables for the model relied on their ability to improve the general model. The results of the multinomial logistic regression were expressed as relative risk ratio (RRR) for each variable with its corresponding 95% confidence interval (CI) and p values were calculated with an alpha level of 0.05.

## Results

### Background characteristics and use of malaria prevention methods

The characteristics of the study population and enrollment criteria are presented in Table 1 and Fig. 1. A total of 4616 women who had a live birth within the 2 years preceding the survey and who attended antenatal care at least once during their most recent pregnancy or pregnancies (within 2 years of the survey). Seventy-percent of the sampled women were aged between 20 and 34 years, and less than 10% were aged below 20. Few pregnant women reported secondary or higher education (12.7%) and 65% reported having never attended school. Socioeconomic characteristics indicate that most of the women lived in the rural areas of Senegal at the time of the survey. Almost all women who had a live birth in the 2 years preceding the survey had their first antenatal visit at or before 6 weeks of pregnancy (98%) and less than half of the pregnant women made four or more antenatal visits (47.4%). For one quarter (25.60%) of the sampled women, the pregnancy was unintended.

**Table 1:** Demographic characteristics of the study participants

Variables	Characteristics N	%
Age (years)		
< 20	405	8.78
20–34	3347	72.50
35–49	864	18.73
Mother’s education level		
No education	3037	65.79
Primary	992	21.49
Secondary and higher	587	12.72
Place of residence		
Urban	1827	39.58
Rural	2789	60.42
Wealth index quintile		
Poorest	1037	22.47
Poorer	1014	21.98
Middle	943	20.42
Richer	826	17.89
Richest	791	17.24
Employment status		
Not working	2664	57.72
Working	1951	42.28
Timing of first ANC visit (weeks)		
≤ 6	4525	98.03
> 6	91	1.97
Number of ANC visits		
4+	2189	47.43
1–3	2427	52.57
Pregnancy intention		
Unintended	1176	25.60
Intended	3418	74.40

*%* Proportion

### Coverage of malaria protection in pregnant women

Less than half of the pregnant women reported using ITNs (46.84%); however, 80.35% reported taking IPTp during their last pregnancy. The highest ITN coverage was found in Ziguinchor (65%). While Dakar had the lowest use of ITNs during pregnancy followed by Kaffrine with 23.3 and 32%, respectively. The highest observed coverage of IPTp was 91.7% in Diourbel and the lowest levels were found in Tambacounda. Nationally, the optimal malaria prevention methods (combined use of ITNs and IPTp) were used by only 37.51% of pregnant women who had a live birth within 2 years preceding the survey.

### Bivariate analysis

To reveal factors associated with the uptake of malaria prevention methods in pregnancy, a bivariate analysis comparing the patient demographics with the outcome of none, partial, or optimal uptake (Table 2) was conducted. Use of both ITNs and IPTp was not significantly different among women of different age groups (p value 0.108). However, the uptake was higher among women in the middle and the richer wealth quintile. Interestingly the most significant difference was observed between the highest wealth quintile and partial uptake. Women who attended ANC visits at least before 6 weeks were more likely to use both ITNs and IPTp than women who sought their first antenatal care after 6 weeks of pregnancy (p value 0.033). Similarly, uptake prevention methods were significantly higher in pregnant women living in regions like Diourbel, Saint Louis, Thies, Louga, Fatick and Matam as compared to women who lived in Dakar at the time of the survey (p value < 0.001).

**Table 2:** Bivariate analysis of uptake of malaria prevention methods during pregnancy in Senegal

Characteristics	Number of respondents	None	Partial (IPTp or INT)	Optimal use (IPTp and INT)	p value
Age (years)					
< 20	405	13.2	53.4	33.8	0.108
20–34	3346	10.2	53.01	36.8	
35–49	865	9.4	48.5	41.8	
Mother’s education level					
No education	3037	11.6	51.4	37.5	0.272
Primary	992	8.4	51.7	39.8	
Secondary and higher	587	9.7	56.7	33.6	
Place of residence					
Urban	1827	8.4	55.2	36.5	0.102
Rural	2789	11.6	50.2	38.2	
Wealth index quintile					
Poorest	1037	15.7	53.38	30.94	
Poorer	1014	10.68	50.48	38.84	< 0.001
Middle	943	5.86	47.94	46.2	
Richer	826	9.42	50.16	40.42	
Richest	796	9.07	59.88	31.05	
Employment status					
Not working	2664	10.67	51	38.5	0.346
Working	1952	9.84	54	36.21	
Number of ANC visits					
1	2426	11.63	52.1	36.3	0.076
4+	2189	08.87	52.26	38.8	
Pregnancy intention					
Unintended	1176	0.08	0.51	0.40	0.076
Intended	3418	0.11	0.52	0.36	
Region					
Dakar	750	78.4	514.8	156.87	
Ziguinchor	146	11.6	39.3	49.08	
Diourbel	480	3.76	45.7	50.83	
Saint-Louis	424	5.98	47.1	46.95	< 0.001
Tambacounda	243	23.7	51.4	24.9	
Kaolack	485	14.68	53.6	31.71	
Thies	721	5.78	54.2	40.04	
Louga	277	5.89	46.6	47.71	
Fatick	227	15.86	38.4	55.97	
Kolda	238	18.57	49.4	32.09	
Matam		7.33	42.4	50.23	
Kaffrine	226	22.45	55.15	22.40	
Kedougou	63	17.87	48.8	33.3	
Sédhiou	137	12.35	49.6	38.02	

*IPTp* intermittent preventive treatment in pregnancy, *ITN* insecticide treated net, *ANC* antenatal care

p value calculated using Pearson’s Chi square test or Fisher’s exact test where Chi square assumptions were not met

### Multinomial logistic regression analysis

Results from multinomial logistic regression are described in Table 3. The relative risk of using optimal malaria prevention methods (IPTp and ITNs) was significantly higher among women who were aged 35–49 years compared to women younger than 20 years old (RRR = 1.62, 95% CI 1.04–2.52, p value 0.043). Uptake of malaria prevention methods (IPTp and ITNs) was 3.56 times more likely among women classified in the middle wealth quintile than women classified in the poorest quintile (RRR = 3.56, 95% CI 2.21–5.75, p value 0.003); however, this relationship declined with increased wealth as uptake of optimal malaria prevention methods (IPTp and ITNs) was almost two times higher among women in the richest wealth quintile than women classified in the poorest quintile (RRR = 1.93, 95% CI 0.94–3.98, p value 0.825).

**Table 3:** Multivariate analysis of uptake of malaria prevention methods during pregnancy in Senegal

Variables	IPTp uptake and ITN usage			
	Partial versus none		Optimal versus none	
	RRR	95% CI (p value)	RRR	95% CI (p value)
Age (years)				
< 20 (reference)	1.00			
20–34	1.22	0.8–1.8 (0.420)	1.36	0.93–1.99 (0.107)
35–49	1.18	0.70–2.02 (0.663)	1.62	1.04–2.52 (0.043)
Place of residence				
Urban (ref)	1.00			
Rural	0.81	0.48–1.3 (0.214)	0.95	0.52–1.71 (0.093)
Wealth index quintile				
Poorest (ref)	1.00		1.00	
Poorer	1.27	0.9–1.8 (0.727)	1.72	1.13–2.60 (0.188)
Middle	2.01	1.4–2.9 (0.145)	3.56	2.21–5.75 (0.003)
Richer	1.19	0.60–2.2 (0.254)	1.93	0.94–3.98 (0.825)
Richest	1.43	0.71–2.8 (0.526)	1.51	0.67–3.40 (0.957)
Number of ANC visits				
4+	1.25	0.90–1.74 (0.076)	1.41	1.03–1.92 (0.049)
1	1		1	
Pregnancy intention				
Unintended (ref)	0.74	0.52–1.06 (0.076)	0.68	0.47–0.98 (0.040)
Intended	1.00		1.00	
Region				
Dakar (reference)	1.00		1.00	
Ziguinchor	0.42	0.20–0.90 (0.024)	2.21	0.85–5.80 (0.109)
Diourbel	2.20	0.90–5.00 (0.059)	10.5	3.38–32.9 (0.001)
Saint-Louis	1.26	0.71–2.15 (0.421)	5.30	2.20–13.1 (0.001)
Tambacounda	0.41	0.19–0.70 (0.030	0.87	0.33–2.19 (0.784)
Kaolack	0.64	0.28–1.27 (0.128)	1.40	0.60–3.80 (0.410)
Thies	1.43	0.56–3.50 (0.460)	4.04	1.24–12.8 (0.018)
Louga	1.33	0.61–2.87 (0.462)	6.00	2.27–16.0 (0.001)
Fatick	1.08	0.53–2.20 (0.824)	7.90	2.82–22.0 (0.001)
Kolda	0.42	0.21–0.83 (0.013)	1.50	0.57–3.84 (0.417)
Matam	1.02	0.46–2.26 (0.947)	5.52	2.01–15.4 (0.001)
Kaffrine	0.42	0.21–0.83 (0.013)	0.93	0.33–2.60 (0.895)
Kedougou	0.41	0.18–0.92 (0.031)	1.40	0.46–4.24 (0.542)
Sédhiou	0.61	0.31–1.20 (0.176)	2.47	0.95–6.40 (0.062)

*Partial* either received IPT or ITNs, *Optimal* combined uptake of ITNs and IPTp, *None* neither IPT nor ITNs, *RRR* relative risk ratio, *95% CI* 95% confidence interval

The number of antenatal care visits attended was also significant predictor of optimal or partial uptake of malaria prevention methods among pregnant women during pregnancy. Women who completed four antenatal visits or more were 1.46 times more likely to have optimal uptake of prevention methods during pregnancy (RRR = 1.41, 95% CI 1.03–1.92, p value 0.049). Likewise, women who attended four antenatal visits or more were 1.25 times more likely to use partial prevention methods (IPTp or ITNs) during pregnancy. Women whose pregnancy was unintended were less likely to use optimal prevention methods than those who planned their pregnancy (RR = 0.68, 95% CI 0.47–0.98, p value 0.040). There were regional differences in uptake of malaria prevention measures. Pregnant women living in regions like Diourbel, Saint-Louis, Thies, Louga, Fatick and Matam were more likely to use both IPTp and ITNs compared to those living in Dakar.

## Discussion

In Senegal, malaria represents a serious public health threat and a leading cause of mortality among children under 5 years of age. The Senegalese National Malaria Control Programme (NMCP) Strategic Plan has set ambitious malaria pre-elimination goals aiming to reduce malaria related mortality to a level close to zero by 2020 [15]. In this context, there is a need to understand factors that influence suboptimal uptake of malaria prevention methods in high-risk groups such as pregnant women. In this study, the effect of different variables on the uptake of malaria prevention methods (IPTp and ITNs) were investigated from a nationally representative dataset. The results of this study show that less than half (< 50%) of women with a recent pregnancy reported use of both IPTp and ITNs during their last pregnancy and nearly half of the participants reported partial use (either IPTp or ITNs) during their last pregnancy. Despite governmental and international donor efforts, the target set by the NMCP and President’s Malaria Initiative of at least 80% ITN coverage in the population and least 80% coverage of IPTp among pregnant women in Senegal has yet to be achieved [12].

While the results of this study reveal that the Senegal has yet to achieve the target described within the National Strategic Plan against malaria [15], the level of uptake of IPTp and ITNs in Senegal are higher in the DHS 2013–2014 compared to previous years according to the results of successive Demographic and Health Surveys: in 2009, the use of IPTp was 12%, and in 2010 it increased to 39% [25]. This finding of lower than target uptake of IPTp and ITNs is in contrast to the high antenatal clinic attendance rate of > 90% nationally. As such, this study confirms similar studies in sub-Saharan Africa which demonstrate a large discordance between frequency of antenatal care visit attendance and use of malaria prevention methods [26, 27]. Since IPTp and ITNs should be given free during antenatal care visits, a possible explanation of the discrepancy between high ANC coverage and suboptimal use of malaria prevention methods is the occurrence of drug or net stock-out at the district or regional level [3]. Additionally, although this study did not specifically look at the provider of the health care system, the huge gap between the rate of antenatal care and the optimal use of malaria prevention methods suggests that there might be other factors related to delivery at the health care facility as well as socioeconomic and individual behavior factors [28–30]. This may suggest the training with more formative supervision of provider practices in the delivery of protective measures against malaria alongside with increasing health promotion activities at the community level on the importance of mothers’ use of IPTp and ITNs [31]. Similar studies in sub-Saharan Africa have demonstrated poor adherence of health workers to provision of IPTp and training them with simplified IPTp messages may be a key strategy in malaria control programme targeting malaria prevention in pregnancy [32, 33]. Previous studies have shown that health care worker training to increase awareness on the importance of ANC attendance are key factors affecting the delivery, access, and use of interventions to prevent malaria in pregnancy in sub-Saharan Africa [30]. Such approaches are planned as part of the NMCP’s “IPTp relaunch plan,” in an effort to maximize ANC visits as opportunities for malaria prevention in pregnancy.

There were large discrepancies between uptake of optimal malaria prevention methods by region. These findings reflect more geographical differences and disparities in the uptake of malaria prevention methods during pregnancy as women living in the regions like Matam, Thies, Louga, Fatick, Diourbel and Saint-Louis were more likely to use optimal prevention methods compared to pregnant women in Dakar. The discrepancies between regions may reflect inequities into access to health care. Place of residence has been previously associated with discrepancies in access and utilization of health care prevention programmes, such as malaria and tuberculosis programmes [20-37-38]. This situation might be explained by the level to which the policies regarding free distribution of IPTp and ITNs have been implemented across the country, especially in the rural areas, but less so in affluent Dakar. Additionally, it is also possible that in some regions, particularly in the rural South of Senegal, due to different factors (level of malaria transmission, rainfall, and efficacy of malaria case management in the health services) women might be more likely to use malaria prevention methods compared to women living in Dakar, the urban capital city of Senegal [33]. The findings from this study are similar to a study in Ethiopia who found an association between the type of place of residence and ITN usage among pregnant women [34].

Women aged 35–49 years were more likely to make use of optimal prevention methods during pregnancy compared to women younger than 20 years of age. This observation may be because young adolescent women are least likely to have a prior pregnancy and are less likely to have previously received information and malaria prevention methods. Further, older women have more knowledge and experience regarding pregnancy and risk of malaria and may, therefore, be more likely to use preventive measures during pregnancy.

Additionally, women classified in the middle wealth quintile were more likely to use malaria protection than women classified in the poorest quintile. These data suggest that even with scale-up of malaria control interventions, present distribution strategies are still not reaching the needs of some of the most vulnerable groups, including the poor. The fact that women from poorer households are disadvantaged in the context of free distribution of IPTp and ITNs [3, 17, 28–33, 35] has been previously documented in studies from Kenya, Senegal and Uganda. In a previous study from DHS data in Senegal, Faye et al. [17] showed that the fact that women have to pay in order to have access to health care centre (where they would receive the freely distributed IPTp and ITN) can negatively impact their access to a health service interventions and tools for malaria prevention in Senegal [17]. Women from poorer households may be faced with long queues at antenatal clinics, as well as transport costs, the ticket to the entry to the clinics which may hinder their access to the freely administered IPTp and ITNs during pregnancy. Hill et al. [30] systematically examined both supply and demand factors associated with low IPTp uptake and low ITN use. In some countries, poorer women, those with no education, or those living in rural areas were significantly less likely to receive IPTp [35].

The use of health survey data can be a powerful tool to inform where challenges remain in current prevention strategies. However, the findings may not be generalizable to all of Senegal. First, the variable used to assess use of ITNs during pregnancy was the self-reported sleeping under an ITN (and not during pregnancy, but the night prior to the survey, with the assumption being that this would be the same as during pregnancy). Some women may be tempted to overestimate their bed-net use as pregnancy is a sensitive issue [34]. Alternatively, this phrasing in the DH survey question could result in misclassification bias as it is possible that women used ITNs during pregnancy, but did not use them the night prior to the survey. Secondly, IPTp administration was assessed only for the proportion of women who had a live birth within the 2 years preceding the survey. Therefore, women with interrupted pregnancy, without a live birth, or women who were pregnant at the time of the survey were not included in the analysis. Thus, there might be a potential for selection bias and the results may not be fully representative of the population of pregnant women in Senegal. However, even with these limitations, the analysis of National Survey Data provides important insights into the factors influencing uptake of malaria prevention methods and can be useful in guiding policy implementation strategies.

## Conclusions

This study has shown important factors that influence the uptake of malaria prevention methods during pregnancy in Senegal. It is imperative that these factors be considered when designing and implementing policies aimed at optimizing malaria prevention strategies in pregnancy in Senegal. Free distribution of IPT-SP and insecticides treated nets within communities and health centres should continue and targeted preventive efforts should be directed to regions or rural areas where optimal use of malaria prevention methods is currently very low. Additionally, specific attention should be paid to improving early and full antenatal clinic attendance at all scheduled visits during pregnancy, and in maximizing education and distribution at these visits as they can provide an important capture point for malaria prevention services.

## Abbreviations

ANC: antenatal care
ANSD: Agence Nationale de la Statistique et de la Démographie
CI: confidence interval
DHS: Demographic and Health Survey
ITNs: insecticide-treated nets
IPTp: intermittent preventative therapy during pregnancy
MCP: malaria control programme
MIS: malaria indicator survey
PAM: pregnancy associated malaria
PSUs: primary sampling units
RRR: relative risk ratio
SP: sulfadoxine–pyrimethamine
WHO: World Health Organization
